# Fibroblast growth factor 23‐mediated regulation of osteoporosis: Assessed via Mendelian randomization and in vitro study

**DOI:** 10.1111/jcmm.18551

**Published:** 2024-07-25

**Authors:** Xiang Zhang, Jin Xu

**Affiliations:** ^1^ Key Laboratory of Endocrine Glucose & Lipids Metabolism and Brain Aging, Ministry of Education; Department of Endocrinology Shandong Provincial Hospital Affiliated to Shandong First Medical University Jinan Shandong China; ^2^ Shandong Key Laboratory of Endocrinology and Lipid Metabolism Jinan Shandong China; ^3^ Shandong Institute of Endocrine and Metabolic Diseases Jinan Shandong China; ^4^ “Chuangxin China” Innovation Base of stem cell and Gene Therapy for endocrine Metabolic diseases Jinan Shandong China; ^5^ Shandong Engineering Laboratory of Prevention and Control for Endocrine and Metabolic Diseases Jinan Shandong China; ^6^ Shandong Engineering Research Center of Stem Cell and Gene Therapy for Endocrine and Metabolic Diseases Jinan Shandong China

**Keywords:** fibroblast growth factor 23, fibroblast growth factor receptor‐1, Mendelian randomization, osteoporosis, α‐Klotho

## Abstract

Despite numerous investigations on the influence of fibroblast growth factor 23 (FGF23), α‐Klotho and FGF receptor‐1 (FGFR1) on osteoporosis (OP), there is no clear consensus. Mendelian randomization (MR) analysis was conducted on genome‐wide association studies (GWASs)‐based datasets to evaluate the causal relationship between FGF23, α‐Klotho, FGFR1 and OP. The primary endpoint was the odds ratio (OR) of the inverse‐variance weighted (IVW) approach. Furthermore, we stably transfected FGF23‐mimic or siRNA‐FGF23 into human bone marrow mesenchymal stem cells (hBMSCs) in culture and determined its cell proliferation and the effects on osteogenic differentiation. Using MR analysis, we demonstrated a strong correlation between serum FGF23 levels and Heel‐ and femoral neck‐BMDs, with subsequent ORs of 0.919 (95% CI: 0.860–0.983, *p* = 0.014) and 0.751 (95% CI: 0.587–0.962; *p* = 0.023), respectively. The expression levels of FGF23 were significantly increased in femoral neck of patients with OP than in the control cohort (*p* < 0.0001). Based on our in vitro investigation, after overexpression of FGF23, compared to the control group, the BMSC's proliferation ability decreased, the expression level of key osteogenic differentiation genes (RUNX2, OCN and OSX) significantly reduced, mineralized nodules and ALP activity significantly decreased. After silencing FGF23, it showed a completely opposite trend. Augmented FGF23 levels are causally associated with increased risk of OP. Similarly, FGF23 overexpression strongly inhibits the osteogenic differentiation of hBMSCs, thereby potentially aggravating the pathological process of OP.

## 1 INTRODUCTION

Osteoporosis (OP), a metabolic bone disorder, is highly prevalent among middle‐aged and older adults and manifests as bone loss or loss of bone microarchitecture, augmenting bone fragility and fracture risk. Emerging evidence from a meta‐analysis suggests that the global incidence of OP and bone loss, as described by the World Health Organization (WHO), was 19.7% (95% CI: 18.0%–21.4%) and 40.4% (95% CI: 36.9%–43.8%), respectively. Developing countries reported substantially more OP cases (22.1%, 95% CI: 20.1%–24.1%), compared to developed countries (14.5%, 95% CI: 11.5%–17.5%).^1^ Moreover, with an increasing global population, the economic and health burdens of OP are rising rapidly. Fibroblast growth factor 23 (FGF23) is a critical member of the FGF family and its genetic code is found on chromosome 12p13. Its action is mediated by Klotho protein‐enriched sites, following secretion by osteoblasts and osteoclasts. Klotho was recently discovered by Matsumura et al. on the chromosome 13q12. It is composed of five exons and four introns and is primarily expressed in the renal tubules, brain choroid, pituitary gland and parathyroid gland.^2^ FGF23 binds to the fibroblast growth factor receptor (FGFR) with a relatively low affinity and α‐Klotho proteins associate with numerous FGFRs to form a complex that regulates FGF23. α‐Klotho and FGFR are the major modulators of FGF23.^3^ FGF23‐deficient mice experience premature ageing, as evidenced by visible atherosclerosis, bone loss, osteoporosis, soft tissue calcification, emphysema, hypogonadism and generalized organ atrophy. These manifestations mirror those observed in mice with defective α‐Klotho expression.^4^ FGF23 modulates the α‐Klotho/FGFR complex (i.e., FGF23 co‐receptor) to exert its physiological effects. This is likely the reason for the strong similarity between Klotho and FGF23 knockout mice. Osteoblast‐secreting FGF23 uniquely targets FGFR1 in renal tubular epithelial cells to form a complex with Klotho. This complex, in turn, suppresses renal phosphorus reabsorption, controls circulating phosphorus homeostasis and indirectly affects bone mineralization. Under α‐Klotho deficiency, FGF23 affinity for FGFR1 is drastically reduced, which slows down FGFR1 phosphorylation and, in turn, decreases bone matrix mineralization.^5^ Nonetheless, it remains unknown whether the FGF23/α‐Klotho/FGFR1 axis is causally associated with OP development.

Mendelian randomization (MR) is commonly employed for aetiology inference in epidemiological research.^6^ With recent advancements in MR research methods, MR is increasingly used to examine the pathogenic relationships between complicated diseases. Here, we employed two‐sample MR analysis and cellular experimental verification to assess the involvement of the FGF23/α‐Klotho/FGFR1 axis in OP development.

## 2 METHODS

### 2.1 Study design

**FIGURE 1:**
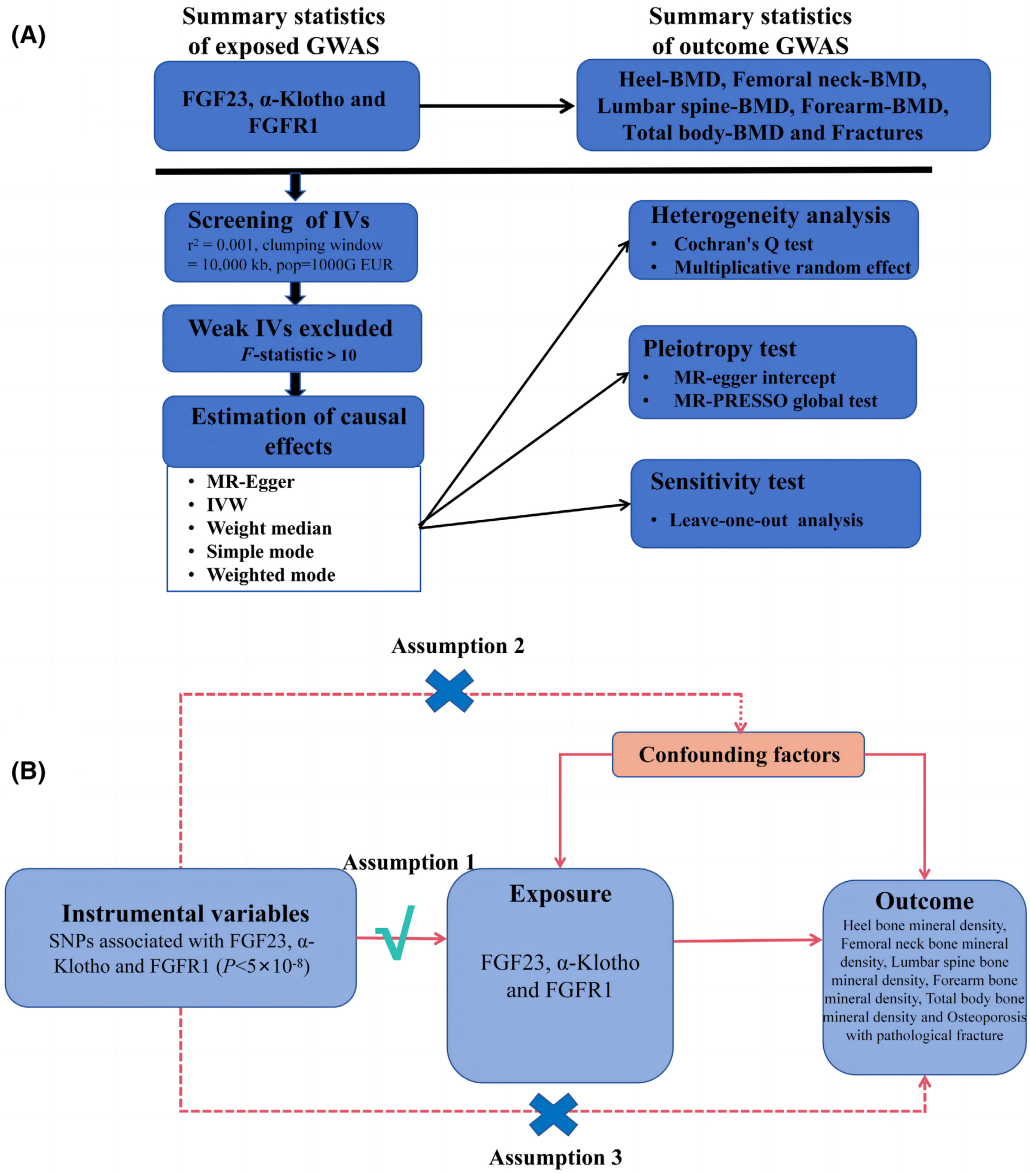
(A) Flow diagram of two‐sample Mendelian randomization analysis; (B) Schematic representation of two‐sample Mendelian randomization.

This research employed two‐sample MR analysis to assess the causal link between serum FGF23, α‐Klotho and FGFR1 levels and six distinct OP outcomes (measured using heel bone mineral density (BMD), femoral neck BMD, lumbar spine BMD, forearm BMD, total body BMD and osteoporosis with pathological fracture) using publicly available data from the genome‐wide association study (GWAS) database (Figure 1A). This investigation employed three primary assumptions^7^: (1) there is a strong correlation between genetic variables and exposure, (2) variables regulate outcomes based on their exposure, and (3) variables do not regulate outcomes based on exposure and confounding factors (Figure 1B).

### 2.2 Instrumental SNPs selection

We selected FGF23‐related instrumental variables (IVs) from a GWAS meta‐analysis of 16,624 European descendants. Among those excluded from analysis were subjects with an estimated glomerular filtration rate (eGFR) <30 mL/min per 1.73 m^2^.^8^ The following seven patient populations were included in the meta‐analysis: Atherosclerosis Risk in Communities Study (ARIC, *n* = 8594),^9^ Indiana Sisters Study (*n* = 1128),^10^ Osteoporotic Fractures in Men Study‐Goteborg (MrOS GBG, *n* = 937),^11^ Multi‐Ethnic Study of Atherosclerosis (MESA, *n* = 2163),^12^ MrOS Malmo (*n* = 894),^11^ Osteoporosis Prospective Risk Assessment Study (OPRA, *n* = 920)^13^ and Cardiovascular Health Study (CHS, *n* = 1988).^14^ Intact FGF23 ELISA (Kainos Laboratories, Inc., Tokyo, Japan) was used in five of seven patient populations (ARIC, Indiana Sisters, MrOS GBG, MrOS Malmo and MESA). For the remaining two patient populations (CHS and OPRA), a C‐terminal ELISA (Immutopics, San Clemente, CA, USA) was employed. Overall, 73% (13,716/16,624) of participant FGF23 were detected using intact FGF23, whereas 27% (2908/16,624) were detected using C‐terminal ELISA. We picked six leading single‐nucleotide polymorphisms (SNPs) from all over the genome that closely correlated with serum FGF23 content using criteria *p* < 5 × 10^−8^ (Table S1). The serum FGF23 units were measured in pg/ml, log‐transformed and the β‐estimates were regarded as the relative difference in FGF23 concentration per minor allele.^8^ The SNPs were normalized according to patient age, sex, study site and principal ancestral components, and they explained 3.0% of the differences in inter‐person serum FGF23 contents.^8^ The aforementioned SNPs resided in various genes and were not in linkage disequilibrium (LD), as evidenced by the results from the LDlink website (https://ldlink.nci.cih.gov/).^15^

Following data integration from the Ludwigshafen Risk and Cardiovascular Health^16^ (LURIC, *n* = 2234) and Avon Longitudinal Study of Parents and Children mothers (ALSPAC, *n* = 2142),^17^, ^18^ there were 4376 European descendants with α‐Klotho data. The α‐Klotho content from both cohorts was detected in plasma samples using a human serum α‐Klotho assay kit (Immuno‐Biological Laboratories Co., Ltd, Japan).^19^ The minimum detection limit was 6.15 pg/mL, with a detection range of 93.75–6000 pg/mL. The variation coefficient was 11.4% at 165.47 pg/mL and 2.9% at 2903.01 pg/mL. Patient sex, age and the first eight major ancestral components (PCs) served as covariates in LURIC, whereas patient sex, age and the first 10 PCs served as covariates in ALSPAC. A Z‐score test, which evaluates the average association estimates between cohorts, was used to assess heterogeneity. Subsequently, we selected six SNPs from across the genome that most closely correlated with serum α‐Klotho content using the criteria *p* < 5 × 10^−8^ (Table S2). These six SNPs explained 7.68% of the difference in serum α‐Klotho content, and they resided in distinct gene regions and not in LD.

To assess genome‐wide relationships, we examined 10.2 million genotyped or imputed autosomal and X‐chromosomal genetic variants using minor allele frequency (MAF) of >1% among 10,708 subjects in the Fenland study involving 4775 proteins.^20^ Subsequently, we selected five SNPs from across the genome that closely correlated with serum FGFR1 content using criteria *p* < 5 × 10^−8^, following adjustments for patient age, sex, study site and major ancestral components (Table S3). The F‐statistic was computed for individual SNP strength evaluation using the equation F = R^2^ (N‐2)/(1‐R^2^), where R^2^ represents the proportion of variance described by IV and N indicates the sample size.

### 2.3 Outcome data sources

We acquired data for six OP outcomes from the GWASs database examining patient populations of European ancestry under the category of Genetic Factors of Osteoporosis (GEFOS) (http://www.gefos.org) and the UK Biobank Consortium. Dual‐energy X‐ray absorptiometry was used to determine and/or detect heel BMD, femoral neck BMD, lumbar spine BMD, forearm BMD, total body BMD and OP with pathological fractures. All studies received ethical approval from the participating institutions. Table S4 presents an exhaustive list of information on the analysed samples. Each GWAS was approved by its corresponding Ethics Committee and these data can be used without restriction.

### 2.4 MR analysis

Using MR analyses, namely inverse‐variance weighted (IVW), weighted median (WM), MR‐Egger, simple mode and weighted mode, we determined a causal relationship between the FGF23/Klotho/FGFR1 axis and OP. Our primary data came from the IVW evaluation, and the individual SNP Wald estimates were presented in a meta‐analysis.^21^ Additional evidence was obtained using MR‐Egger,^22^ WM analyses,^23^ which, although less efficient, provided strong estimates under a broader range of conditions. A power calculation was performed using mRnd (https://cnsgenomics.com/shiny/mRnd/), developed by Brion et al.^24^ Additionally, we used sensitivity analyses to examine possible pleiotropy and heterogeneity, and heterogeneity reporting was done using Cochran's test (*p* < 0. 05). It should be noted that heterogeneity does not detract from the MR estimations as we used a random‐effect model for the IVW method. MR‐Egger regression intercepts were used to assess horizontal polytropy (*p* < 0. 05).^25^ The MR‐pleiotropy residual sum and outlier (MR‐PRESSO), a newly proposed MR method, is a variation on the IVW method. MR‐PRESSO global test is used to assess the presence of overall horizontal pleiotropy.^26^ IV performance was examined using a leave‐one‐out approach.

### 2.5 Clinical sampling

Between November 2023 and April 2024, 40 hip arthroplasties were conducted on patients with primary osteoporotic fractures and non‐osteoporotic fractures at Shandong Provincial Hospital. Bone tissue samples from non‐osteoporotic donors were acquired from femoral heads following total hip arthroplasty prompted by osteoarthritis and/or hip dysplasia. Conversely, bone tissue samples from patients with osteoporosis were obtained from femoral necks following low‐energy fracture of the femoral neck. Additional criteria utilized to confirm primary osteoporosis in these donors included vertebral fractures and advanced age. The bone tissue from the femoral neck of these patients was collected, ground, rinsed with phosphate‐buffered saline (PBS) and stored at ˗80°C. The study included middle‐aged and older men aged over 55 years. Informed consent was obtained from all patients. The characteristics of patients and the general subjects are presented in Table S5. This study was approved by the Ethics Review Committee of Shandong Provincial Hospital (SWYX: NO. 2023‐572).

### 2.6 Cell culture and osteogenic differentiation

We acquired human bone marrow mesenchymal stem cells (hBMSCs) from Procell (Wuhan, China) and grew them in α‐minimum essential medium (α‐MEM; Procell, Wuhan, China) with 10% fetal bovine serum (FBS; Gibco, NY, USA), 2 mM L‐glutamine (Sigma‐Aldrich, MO, USA), and 1% penicillin and streptomycin (Gibco). To initiate osteogenic differentiation, hBMSCs were stimulated with fresh osteogenic medium (0.1 mM dexamethasone, β‐glycerophosphate (10 mM) and ascorbic acid (0.1 mM) (Procell, Wuhan, China)) for 14 days in a humid chamber at 5% carbon dioxide (CO_2_) at 37°C.

### 2.7 Plasmid incorporation

SiRNA‐FGF23, OE‐FGF23 and FGF23‐NC were prepared by Tsingke Biotech Co. Ltd. (Beijing, China). Following dilution to a final concentration of 500 nM, siRNA‐FGF23, OE‐FGF23 and FGF23‐NC were incorporated into hBMSCs, whereas control cells were not incorporated.

### 2.8 Cell proliferation assay

Plasmid‐incorporated hBMSCs (48 h) were evaluated for cellular proliferation using Cell Counting kit‐8 (CCK‐8; Abcam, Cambridge, UK). Briefly, 10 μL of reagent was added to the cells at 24, 48, 72 and 96 h, with subsequent 2 h at 37°C. Optical density was measured at 450 nm using a microplate reader (Devices Spectra Mani3X, Molecular, USA).

### 2.9 Alkaline phosphatase (ALP) staining (Azo coupling method)

ALP activity is strongly proportional to osteogenic differentiation; that is, enhanced ALP activity represents augmented osteogenic differentiation. We cultured hBMSCs for 14 days in induction medium prior to removal and immobilization in 4% paraformaldehyde (PFA; Servicebio, Wuhan, China). ALP staining was performed using an ALP staining kit (Mlbio, Shanghai, China) and associated directions. Finally, the images were captured using an inverted phase‐contrast microscope (Olympus, Tokyo, Japan).

### 2.10 Alizarin red staining

We assessed hBMSCs mineral deposition following osteogenic induction using Alizarin red staining. Following a 21‐day culture in induction medium, hBMSCs were fixed in 4% PFA prior to 1% ARS staining (Procell, Wuhan, China) at 25°C and imaged capture under an inverted phase contrast microscope (Olympus, Japan).

### 2.11 Quantitative real‐time polymerase chain reaction (qRT‐PCR)

Total RNA was extracted from cells using the Eastep Super RNA Extract Reagent Kit (Tiangen, Beijing, China) and associated directions, 0.5 μg of RNA was converted into cDNA using Applied Biosystems. Subsequently, we used Power SYBR Green PCR Master Mix (Vazyme, Nanjing, China) and Quantstudio 6 flex (Applied Biosystems) to conduct the qRT‐PCR assay. Relative FGF23 expression was determined by CT normalization of the internal control, glyceraldehyde‐3‐phosphate dehydrogenase (GAPDH). The primer sequences for FGF23, RUNX2, OCN, OSX and GAPDH are summarized in Table S6.

### 2.12 Western blotting

Following total protein extraction using the radioimmunoprecipitation assay buffer (Biocolor, Shanghai, China), 20 μg protein was electrophoresed in 10% sodium dodecyl sulphate prior to transfer onto nitrocellulose membrane. Subsequently, the membrane was rinsed in TBS with 0.05% Tween 20 (TBST) prior to a 1‐h blocking in 5% skim milk at 25 C, overnight incubation with anti‐FGF23 (1:1000; Abcam, Cambridge, UK) and anti‐GAPDH (1:3000; Abcam) at 4°C, heating at 25°C, and a 2‐h incubation with secondary antibody (1:5000; Proteintech, IL, USA). Protein bands were visualized using enhanced chemiluminescence and protein quantification was performed using an Amersham Imager 680 (GE Healthcare, USA).

### 2.13 Statistical analysis

MR analyses utilized ‘TwoSampleMR’ and ‘MR‐PRESSO’ packages in R 4.2.2 (www.r‐project.org), and data are provided as mean effect per one standard deviation (SD) increase of the genetically estimated circulating FGF23, α‐Klotho and FGFR1 contents, as well as the associated 95% confidence intervals (CIs). Statistical significance was set at *p* < 0.05. All data analyses employed GraphPad Prism9, and the results are provided as x ± s. The Students *t*‐test was used to compare differences between two groups, whereas one‐way and two‐way analysis of variance (anova) and Dunnett's or Tukey's multiple comparison tests were used for multiple group comparisons, and *p* < 0.05.

## 3 RESULTS

### 3.1 MR analysis

**FIGURE 2:**
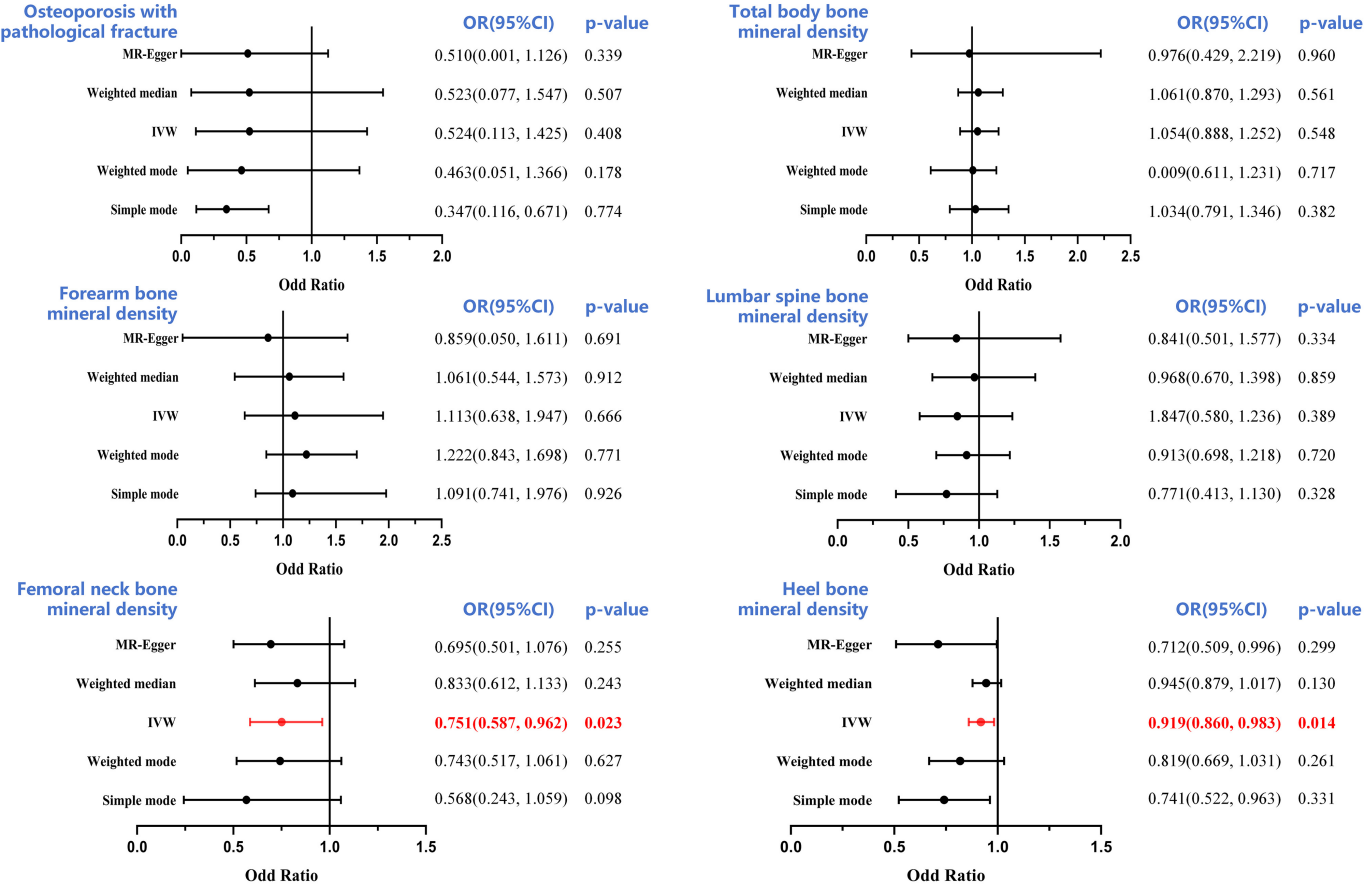
Forest plot to visualize the causal effect of FGF23 on the risk of osteoporosis. BMD, bone mineral density; CI, confidence interval IVW, inverse‐variance weighted.

**FIGURE 3:**
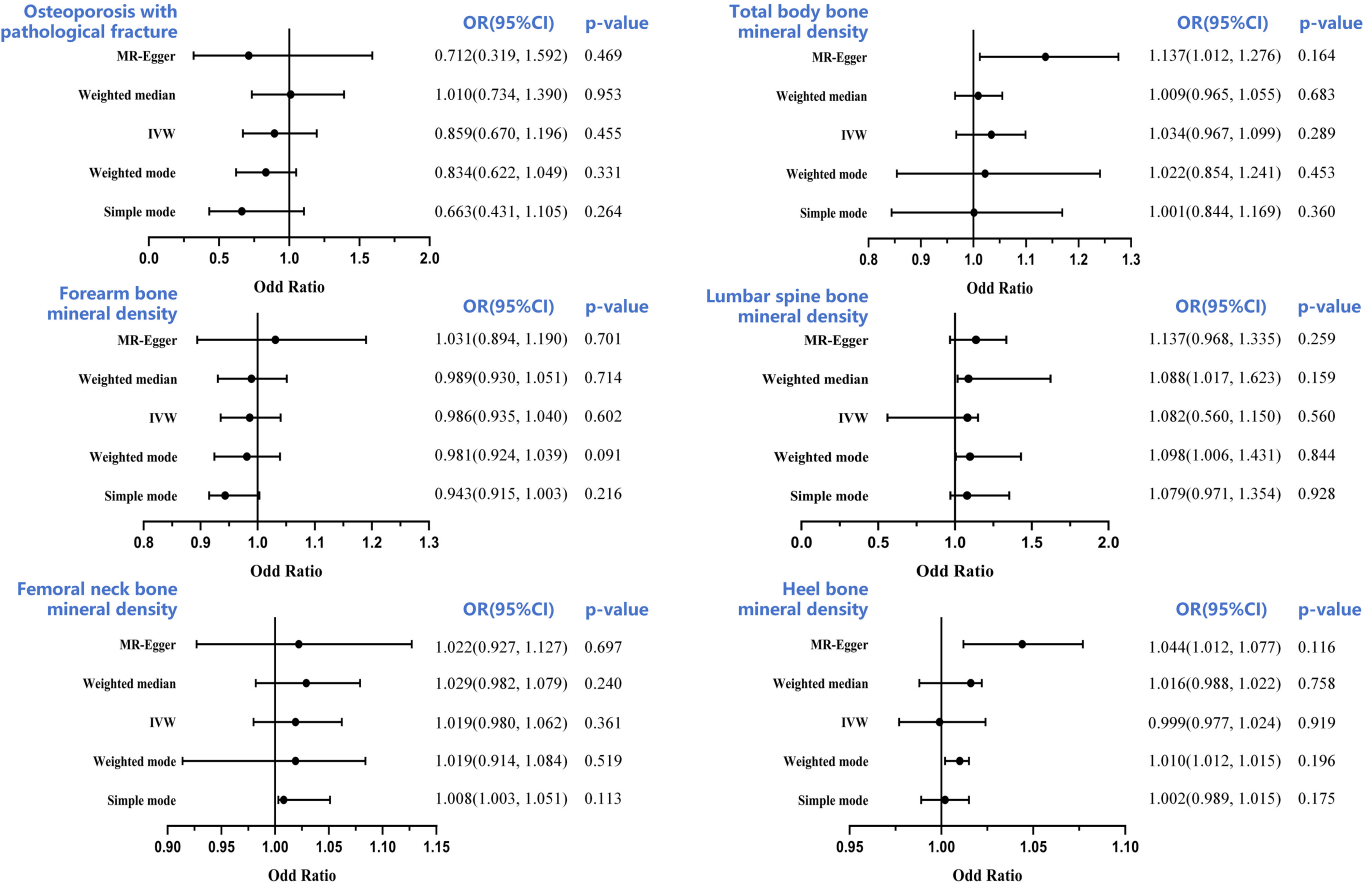
Forest plot to visualize the causal effect of α‐Klotho on the risk of osteoporosis. BMD, bone mineral density; CI, confidence interval IVW, inverse‐variance weighted.

**FIGURE 4:**
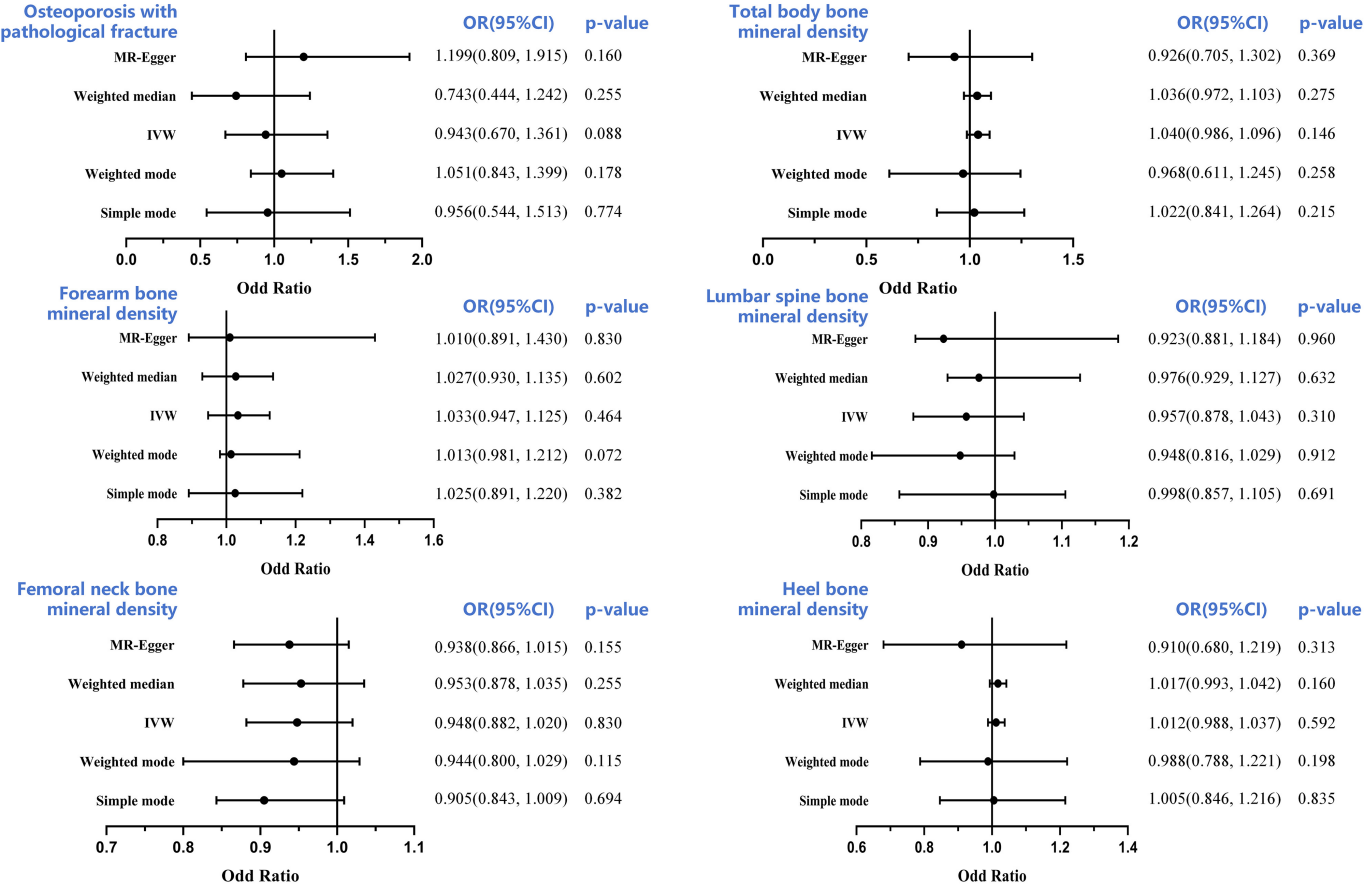
Forest plot to visualize the causal effect of FGFR1 on the risk of osteoporosis. BMD, bone mineral density; CI, confidence interval; IVW, inverse‐variance weighted.

The minimum F values of all IVs were >30, suggesting a lack of a weak IVs bias. Power calculations were conducted according to Brion et al.^24^ Our sample provided sufficient statistical power (>80%) for causal analysis of FGF23, α‐Klotho and FGFR1 on BMDs with a sample size of 426,824, 32,735, 28,498, 21,907, 56,284 and 173,619, respectively. Based on our IVW analyses, serum FGF23 levels were closely correlated with Heel BMD and femoral neck BMD, with ORs of 0.919 (95% CI: 0.860–0.983, *p* = 0.014) and 0.751 (95% CI: 0.587–0.962; *p* = 0.023), respectively (Figure 2). These findings suggest that an increase in circulating FGF23 levels severely decreases Heel BMD and femoral neck BMD, thereby enhancing OP risk. We also observed no causal association between circulating FGF23 levels and lumbar spine BMD, forearm BMD, total body BMD or OP fractures (all *p* > 0.05) (Figure 2). In addition, circulating α‐Klotho and FGFR1 levels were not associated with OP or OP fractures (all *p* > 0.05) (Figures 3 and 4 **)**. All MR‐Egger, WM, simple mode and weighted mode analyses revealed causality comparable to that of the IVW analysis, and these data are provided as additional evidence in Figures 3 and 4. To examine data reliability, we conducted a sensitivity analysis and found no possible horizontal pleiotropy (all *p* > 0.05) (Table S7). Additional sensitivity analyses revealed slight heterogeneity in α‐Klotho‐Heel BMD (*p* = 7.21 × 10^−4^) and α‐Klotho‐total body BMD (*p* = 6.62 × 10^−3^), however, using a random‐effects model of IVW did not alter the conclusions of the prior analyses (all *p* > 0.05) (Table S7). Figures S1 and S2 present the outcomes of the sensitivity analysis, scatter plots, forest plots and funnel plots of the positive causal effects of the FGF23 on the OP‐outcomes. Collectively, these findings suggest that the outcomes of all analyses examining the association between serum FGF23, α‐Klotho and FGFR1 levels and OP and OP fracture risk are reliable.

### 3.2 FGF23 accelerates OP development by suppressing osteogenic differentiation of hBMSCs

**FIGURE 5:**
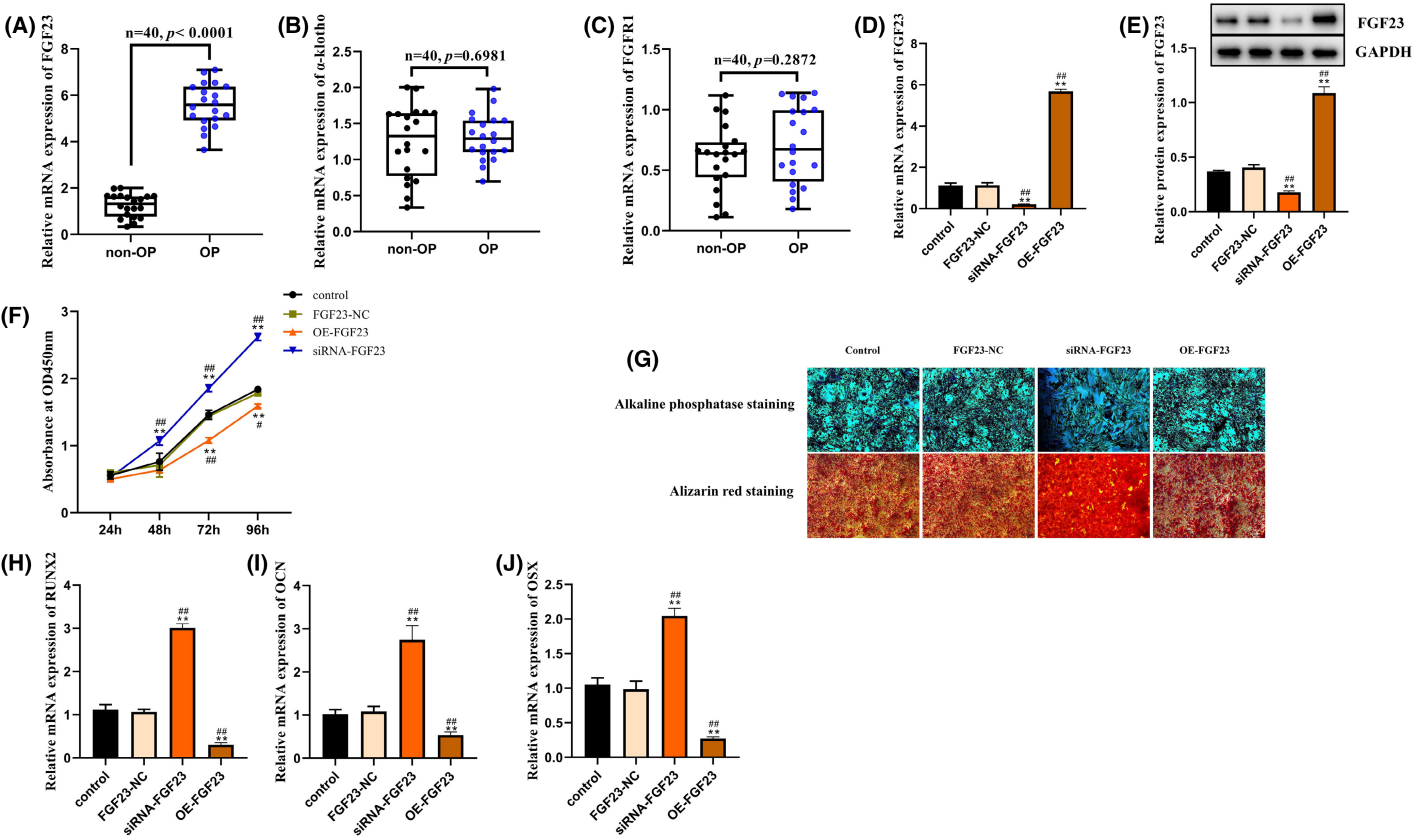
(A–C) Relative FGF23, a‐Klotho and FGFR1 expression in femoral neck of osteoporosis (*n* = 20) or non‐osteoporosis patients (*n* = 20). (D) Relative mRNA expression of FGF23 was determined by qRT‐PCR in hBMSCs transfected with the OE‐FGF23, siRNA‐FGF23 or FGF23‐NC negative control (NC). (E) Relative protein expression of FGF23 was detected by western blotting in hBMSCs transfected with the OE‐FGF23, siRNA‐FGF23 or FGF23‐NC. (F) After 24, 48, 72 and 96 h culture, cell proliferation was detected by Cell Counting kit‐8 (CCK‐8) assay in hBMSCs transfected with OE‐FGF23, siRNA‐FGF23 or FGF23‐NC. (G) ALP and ARS staining were used to detect ALP activity and calcium nodules in hBMSCs transfected with OE‐FGF23, siRNA‐FGF23 or FGF23‐NC, respectively. (H–J) In the hBMSCs transfected with OE‐FGF23, siRNAFGF23 or FGF23‐NC, the expression levels of RUNX2, OCN and OSX were determined by qRT‐PCR. **p < 0.01 compared with control group. ^#^ *p* < 0.05, ^##^ *p* < 0.01 compared with FGF23‐NC group. Control and blank control.

QRT‐PCR revealed that FGF23 expression levels were significantly increased in OP patients, which were four fold higher than those in non‐OP patients (*p* < 0.0001) (Figure 5A). However, there were no significantly changes of α‐Klotho and FGFR1 expression between OP and non‐OP patients (Figure 5B,C). To elucidate the mechanism by which FGF23 regulates hBMSCs differentiation, we incorporated OE‐FGF23 or siRNA‐FGF23 into the hBMSCs. Using qRT‐PCR and western blot assays, we revealed that OE‐FGF23 hBMSCs expressed substantially more FGF23 (approximately 6‐fold) mRNA and protein (approximately 2‐fold) than control hBMSCs, while siRNA‐FGF23 hBMSCs expression of mRNA and protein was significantly inhibited (Figure 5D,E). Next, we explored the alterations in the proliferation of hBMSCs at 24, 48, 72 and 96 h post plasmid incorporation. We found that the optical density of OE‐FGF23 hBMSCs was drastically diminished relative to that of the control hBMSCs (*p* < 0.01). However, the cell proliferation rate was significantly increased in siRNA‐FGF23 group compared with the control group (Figure 5F). After 14 days of osteogenic induction, ALP staining was considerably reduced in OE‐FGF23 hBMSCs compared to that in control hBMSCs. After 21 days of osteogenic induction, alizarin red staining among OE‐FGF23 hBMSCs was also diminished, suggesting impaired calcium deposition relative to control hBMSCs. After silting FGF23 expression, both the activity of ALP and number of calcium nodules of ARS were significantly increased (Figure 5G). Osteogenic differentiation results from several signalling networks and factor activation, among which RUNX2, OCN and OSX are critical transcription factors that stimulate osteoblast differentiation and initiate extracellular matrix calcification.^27^ After 21 days of osteogenic induction, compared to control hBMSCs, RUNX2, OCN and OSX mRNA levels were drastically reduced in OE‐FGF23 hBMSCs, while mRNA levels of RUNX2, OCN and OSX in siRNA‐FGF23 hBMSCs were significantly increased compared with control hBMSCs (Figure 5H–J). Together, these findings suggested that FGF23 overexpression strongly suppressed hBMSCs differentiation into osteoblasts, which, in turn, accelerates OP pathology.

## 4 DISCUSSION

Bone homeostasis is strictly modulated by a delicate balance between osteoclast‐based bone resorption and osteoblast‐based bone synthesis^28^ in a process called bone remodelling. OP occurs once this balance is disrupted, and a major contributor to this disruption is dysregulation of multiple cytokines.^29^ FGF23 is a bone‐derived hormone intricately linked to OP pathology.^30^, ^31^ However, it remains controversial whether the FGF23/α‐Klotho/FGFR1 axis modulates OP development.^32^, ^33^, ^34^, ^35^ Prior investigations have suggested a potential link between FGF23/α‐Klotho/FGFR1 and OP^36^; however, there is no concrete evidence for this link, as prior conclusions have been marked by confounding factors and challenges when conducting large‐scale case–control and cohort investigations.

Here, we systematically examined the link between the FGF23/α‐Klotho/FGFR1 axis and OP development risk using a two‐sample MR investigation. We demonstrated that serum FGF23 was causally correlated with OP; that is, upregulated serum FGF23 levels drastically reduced BMD, which in turn enhanced OP and fracture risks. Unlike other studies, we found no evidence of a causal relationship between α‐Klotho/FGFR1 and OP or fracture risk. MR analysis has certain advantages over observational investigation. First, we selected SNPs that were strongly correlated with exposure factors. We employed large‐sample and multicentre data which were extensively screened for exposure‐ and outcome occurrence‐related confounders, and excluded confounder‐related SNPs to minimize possible horizontal pleiotropy in genetic IVs, while employing MR‐Egger and MR‐PRESSO global test to further examine potential pleiotropy to ensure the outcome reliability.^37^ Furthermore, we used Cochran's Q test to assess IV heterogeneity. In the absence of marked heterogeneity in Cochran's Q test, we performed an unbiased association estimation using an IVW linear regression. In the presence of marked heterogeneity, the IVW model was employed with random effects to ensure the accuracy of the results.^38^ Second, apart from the IVW analysis, we also conducted other sensitivity analyses, such as WM, MR‐Egger and Simple Mode, to further confirm outcome accuracy.^39^ Overall, this study demonstrated a gene‐level correlation between FGF23 levels and the risk of OP.

To date, we have established a strong link between serum FGF23 levels and OP risk at a genetic level. However, only a limited number of studies have explored the effects of localized FGF23 on osteoblasts and the bone microenvironment. FGF23 is synthesized within osteoblasts and coordinates activities involving osteoblasts and osteoclasts. Hypophosphataemia and rickets/osteomalacia typically correlate with enhanced FGF23 levels in multiple hereditary disorders, thus providing clear evidence of a negative association between FGF23 and bone mineralization.^40^, ^41^, ^42^ α‐Klotho, a single‐channel transmembrane protein, serves as an FGF23 co‐receptor, and recent evidence has revealed ubiquitous α‐Klotho expression in osteoblasts of murine skull and long bones.^43^, ^44^ Additionally, other investigations have revealed augmented FGF23 and α‐Klotho transcript levels in piglet epiphyses, bone marrow and tissues adjacent to the epiphysis (e.g. secondary ossification centres), indicating involvement of the mineralization process.^45^ The critical presence of essential Klotho proteins in skeletal tissues indicates a direct function of FGF23 in the bone‐remodelling process.

Subsequently, we examined FGF23‐mediated regulation of osteogenic differentiation of hBMSCs. Following FGF23 overexpression, we revealed that the proliferation rate of OE‐FGF23 hBMSCs was drastically reduced relative to that of control hBMSCs at various time points (24 h, 48 h, 72 h and 96 h) (*p* < 0.01). Upon in vitro osteogenic induction, osteoblasts can proliferate and secrete bone matrix factors, such as collagen, non‐collagenous proteins and bone formation‐related factors. Once mature, differentiated osteoblasts synthesize and release large quantities of ALP. ALP is a well‐established marker of osteoblastic differentiation. Moreover, its activity is somewhat representative of osteoblast differentiation status. Hence, after 14 days of osteogenic induction, ALP staining was performed, as depicted in Figure 5. Relative to control hBMSCs, OE‐FGF23 hBMSCs exhibited markedly diminished ALP staining. ARS is an anthraquinone derivative that is frequently used to assess calcium alterations in tissue sections or cultured cells in vitro. Alizarin Red detects calcium‐containing osteoblasts in hBMSCs induced by calcium chelation to form an orange‐red complex. Hence, they indirectly reflect the status of the induced differentiation. On the 21st day post‐induction, we conducted alizarin red staining to show that relative to the control hBMSCs, calcium deposition was markedly reduced in the OE‐FGF23 hBMSCs. Next examined the FGF23‐mediated effect on osteogenic differentiation of hBMSCs. We revealed that FGF23 strongly diminished RUNX2, OSN and OSX gene expression during bone formation while inhibiting calcium nodule formation. In addition, we provided further evidence by transfecting small interfering RNA to suppress the expression level of FGF23 in hBMSCs. Our results confirmed that after inhibiting the expression of FGF23, the proliferation level of hBMSCs significantly decreased, calcium nodules and ALP activity decreased, and the expression level of key osteogenic differentiation genes also decreased. These results fully demonstrate the important role of FGF23 in the osteogenic differentiation of hBMSCs, that is, overexpression of FGF23 inhibits the osteogenic differentiation process of hBMSCs, leading to OP. However, the relationship between the decrease in the cell proliferation rate of hBMSCs caused by overexpression of FGF23 and the inhibition of the expression level of osteogenic differentiation genes is a complex biological problem. In many cases, these two processes may be related. First, cell proliferation is the process of cell growth and division, and osteogenic differentiation refers to the differentiation of cells into osteoblasts. These two processes are important parts of the growth and development of organisms. In some cases, the decrease in cell proliferation rate may lead to a decrease in the expression level of osteogenic differentiation genes. This is because, due to the decrease in cell proliferation rate, the decrease in osteoblasts leads to a significant decrease in the expression of osteogenic differentiation genes. Therefore, in future research, we need to further detect the level of adipogenic differentiation genes, confirm that in the case of overexpression of FGF23, the osteogenic differentiation of hBMSCs is inhibited, and the level of adipogenic differentiation is increased, to distinguish the impact of cell proliferation level on the differentiation process of hBMSCs. In conclusion, this study validated the notion that FGF23 directly inhibits osteogenic differentiation of hBMSCs in vitro.

The strength of this study lies in the use of MR analysis, which takes advantage of the genetic endowment randomly assigned at conception and examines the lifelong effects of exposure. Therefore, compared to traditional observational studies, it is less susceptible to confounding factors and reverse causality. To our knowledge, this is the first study to explore the causal relationship between FGF23 and OP using MR methods. SNPs closely related to the exposure factors have been strictly screened for subsequent MR analysis. In order to ensure the robustness of the results, we also conducted heterogeneity tests, pleiotropy tests and leave‐one‐out analysis. In addition, by transfecting OE‐FGF23 or siRNA‐FGF23 in vitro to stably express in hBMSCs, the impact of FGF23 on the osteogenic differentiation of hBMSCs was further confirmed, which validates the reliability of the conclusion. However, this study also has some limitations. First, when IVs are chosen to explain only a small portion of the exposure, the presence of weak IVs bias may render MR analyses insufficient for detecting causal effects. Second, to prevent confounding bias caused by horizontal or vertical gene pleiotropy, the MR analysis performed here requires a comprehensive understanding of gene–disease and gene–intermediate phenotype interactions. However, fully controlling these confounding effects is challenging because the functions of these genes are complex and may affect a range of metabolic pathways and other biological activities through poorly understood mechanisms. Third, although the overall sample size used in this study was large, publicly available GWAS datasets were summary statistics rather than individual‐level data, and could not be stratified according to age or other characteristics, thus limiting the evaluation of the relationship between serum FGF23 and BMDs. Fourth, the GWAS data used in this study were all from individuals of European ancestry. Whether the results observed in this study are applicable to other populations remains to be determined. Therefore, future studies using MR analysis to investigate the causal relationship between serum FGF23 levels and OP should include samples from different ethnic groups to increase the generalizability of the results. Although the effect of FGF23 on osteogenic differentiation has been confirmed through in hBMSCs, the lack of in vivo experiments with FGF23‐knockout mice cannot better simulate the in vivo biological environment and verify the feasibility of the research results. In addition, the specific mechanism of FGF23 regulating osteogenic differentiation needs further study. For example, it would be beneficial to explore how FGF23 affects other signalling pathways and how they interact with other factors. Finally, although this study has deeply explored the role of FGF23 in OP, it has not conducted adipogenic induction differentiation research, nor has it verified the specific effects of FGF23 on osteoblasts and osteoclasts.

## 5 CONCLUSION

Herein, we are the first to report a causal link between serum FGF23 contents and BMD levels, which may, in turn, enhance OP risk, using two‐sample MR circulating α‐Klotho and FGFR1 contents showed no association with OP or fracture risk. Using in vitro cellular experiments, we further validated the FGF23‐mediated regulation of bone tissue mineralization, that is, FGF23 strongly suppressed osteogenic differentiation and calcium deposition in hBMSCs. Given this evidence, additional extensive investigations are warranted to elucidate the role and mechanism underlying FGF23 action, particularly in bone mineralization and bone microenvironmental homeostasis. We believe that progress made in this area will greatly benefit OP management.

## AUTHOR CONTRIBUTIONS

**Xiang Zhang:** Conceptualization (equal); formal analysis (equal); investigation (equal); methodology (equal); software (equal); writing – original draft (equal); writing – review and editing (equal). **Jin Xu:** Conceptualization (equal); funding acquisition (equal); investigation (equal); project administration (equal); resources (equal); supervision (equal); validation (equal); visualization (equal); writing – review and editing (equal).

## FUNDING INFORMATION

This work was supported by the National Key Research and Development Program of China (2021YFC2501700 and 2021YFC2501705), Taishan Scholar Construction Project Special Funding (TS201712092) and National key Research and Development Project (2023YFC3504300).

## CONFLICT OF INTEREST STATEMENT

The authors confirm that there are no conflicts of interest.

## Supporting information


Figure S1.

Figure S2.

Table S1.
Table S2.
Table S3.
Table S4.
Table S5.
Table S6.
Table S7.

## Data Availability

The data that support the findings of this study are available from the corresponding author upon reasonable request.

## References

[1] Xiao PL , Cui AY , Hsu CJ , et al. Global, regional prevalence, and risk factors of osteoporosis according to the World Health Organization diagnostic criteria: a systematic review and meta‐analysis[J]. Osteoporos Int. 2022;33(10):2137‐2153. 35687123 10.1007/s00198-022-06454-3

[2] Gutierrez O , Isakova T , Rhee E , et al. Fibroblast growth factor‐23 mitigates hyperphosphatemia but accentuates calcitriol deficiency in chronic kidney disease. J Am Soc Nephrol. 2005;16(7):2205‐2215. 15917335 10.1681/ASN.2005010052

[3] Hao Q , Wang Y , Ding X , et al. G‐395A polymorphism in the promoter region of the KLOTHO gene associates with frailty among the oldest‐old. Sci Rep. 2018;8(1):6735. 29712948 10.1038/s41598-018-25040-4 PMC5928057

[4] Razzaque MS , Lanske B . Hypervitaminosis D and premature aging: lessons learned from Fgf23 and Klotho mutant mice. Trends Mol Med. 2006;12(7):298‐305. 16731043 10.1016/j.molmed.2006.05.002

[5] Olauson H , Vervloet MG , Cozzolino M , Massy ZA , Ureña Torres P , Larsson TE . New insights into the Fgf23‐Klotho axis. Semin Nephrol. 2014;34(6):586‐597. 25498378 10.1016/j.semnephrol.2014.09.005

[6] Sekula P , Del GMF , Pattaro C , et al. Mendelian randomization as an approach to assess causality using observational data. J Am Soc Nephrol. 2016;27(11):3253‐3265. 27486138 10.1681/ASN.2016010098 PMC5084898

[7] Lawlor DA , Harbord RM , Sterne JA , et al. Mendelian randomization: using genes as instruments for making causal inferences in epidemiology. Stat Med. 2008;27(8):1133‐1163. 17886233 10.1002/sim.3034

[8] Robinson‐Cohen C , Bartz TM , Lai D , et al. Genetic variants associated with circulating fibroblast growth factor 23. J Am Soc Nephrol. 2018;29(10):2583‐2592. 30217807 10.1681/ASN.2018020192 PMC6171267

[9] The ARIC investigators . The Atherosclerosis Risk in Communities (ARIC) Study: design and objectives. Am J Epidemiol. 1989;129(4):687‐702. 2646917

[10] Peacock M , Koller DL , Hui S , Johnston CC , Foroud T , Econs MJ . Peak bone mineral density at the hip is linked to chromosomes 14q and 15q. Osteoporos Int. 2004;15(6):489‐496. 15205721 10.1007/s00198-003-1560-7

[11] Mellstrom D , Johnell O , Ljunggren O , et al. Free testosterone is an independent predictor of BMD and prevalent fractures in elderly men: Mros Sweden. J Bone Miner Res. 2006;21(4):529‐535. 16598372 10.1359/jbmr.060110

[12] Bild DE , Bluemke DA , Burke GL , et al. Multi‐Ethnic Study of Atherosclerosis: objectives and design. Am J Epidemiol. 2002;156(9):871‐881. 12397006 10.1093/aje/kwf113

[13] Gerdhem P , Brandstrom H , Stiger F , et al. Association of the collagen type 1 (COL1A 1) Sp1 binding site polymorphism to femoral neck bone mineral density and wrist fracture in 1044 elderly Swedish women. Calcif Tissue Int. 2004;74(3):264‐269. 14595528 10.1007/s00223-002-2159-2

[14] Fried LP , Borhani NO , Enright P , et al. The cardiovascular health study: design and rationale. Ann Epidemiol. 1991;1(3):263‐276. 1669507 10.1016/1047-2797(91)90005-w

[15] Machiela MJ , Chanock SJ . LDlink: a web‐based application for exploring population‐specific haplotype structure and linking correlated alleles of possible functional variants. Bioinformatics. 2015;31(21):3555‐3557. 26139635 10.1093/bioinformatics/btv402 PMC4626747

[16] Winkelmann BR , Marz W , Boehm BO , et al. Rationale and design of the LURIC study—a resource for functional genomics, pharmacogenomics and long‐term prognosis of cardiovascular disease. Pharmacogenomics. 2001;2(1 Suppl 1):S1‐S73. 11258203 10.1517/14622416.2.1.S1

[17] Fraser A , Macdonald‐Wallis C , Tilling K , et al. Cohort Profile: the Avon Longitudinal Study of Parents and Children: ALSPAC mothers cohort. Int J Epidemiol. 2013;42(1):97‐110. 22507742 10.1093/ije/dys066 PMC3600619

[18] Boyd A , Golding J , Macleod J , et al. Cohort Profile: the'children of the 90s'—the index offspring of the Avon Longitudinal Study of Parents and Children. Int J Epidemiol. 2013;42(1):111‐127. 22507743 10.1093/ije/dys064 PMC3600618

[19] Yamazaki Y , Imura A , Urakawa I , et al. Establishment of sandwich ELISA for soluble alpha‐Klotho measurement: Age‐dependent change of soluble alpha‐Klotho levels in healthy subjects. Biochem Biophys Res Commun. 2010;398(3):513‐518. 20599764 10.1016/j.bbrc.2010.06.110 PMC4130489

[20] Pietzner M , Wheeler E , Carrasco‐Zanini J , et al. Mapping the proteo‐genomic convergence of human diseases. Science. 2021;374(6569):eabj1541. 34648354 10.1126/science.abj1541 PMC9904207

[21] Burgess S , Dudbridge F , Thompson SG . Combining information on multiple instrumental variables in Mendelian randomization: comparison of allele score and summarized data methods. Stat Med. 2016;35(11):1880‐1906. 26661904 10.1002/sim.6835 PMC4832315

[22] Bowden J , Del GMF , Minelli C , et al. Assessing the suitability of summary data for two‐sample Mendelian randomization analyses using MR‐Egger regression: the role of the I2 statistic. Int J Epidemiol. 2016;45(6):1961‐1974. 27616674 10.1093/ije/dyw220 PMC5446088

[23] Bowden J , Davey SG , Haycock PC , et al. Consistent estimation in Mendelian randomization with some invalid instruments using a weighted median estimator. Genet Epidemiol. 2016;40(4):304‐314. 27061298 10.1002/gepi.21965 PMC4849733

[24] Brion MJ , Shakhbazov K , Visscher PM . Calculating statistical power in Mendelian randomization studies. Int J Epidemiol. 2013;42(5):1497‐1501. 24159078 10.1093/ije/dyt179 PMC3807619

[25] Burgess S , Thompson SG . Interpreting findings from Mendelian randomization using the MR‐Egger method. Eur J Epidemiol. 2017;32(5):377‐389. 28527048 10.1007/s10654-017-0255-x PMC5506233

[26] Ong JS , Macgregor S . Implementing MR‐PRESSO and GCTA‐GSMR for pleiotropy assessment in Mendelian randomization studies from a practitioner's perspective. Genet Epidemiol. 2019;43(6):609‐616. 31045282 10.1002/gepi.22207 PMC6767464

[27] Mathews S , Bhonde R , Gupta PK , Totey S . Extracellular matrix protein mediated regulation of the osteoblast differentiation of bone marrow derived human mesenchymal stem cells. Differentiation. 2012;84(2):185‐192. 22664173 10.1016/j.diff.2012.05.001

[28] Sakai T , Honzawa S , Kaga M , Iwasaki Y , Masuyama T . Osteoporosis pathology in people with severe motor and intellectual disability. Brain and Development. 2020;42(3):256‐263. 31982226 10.1016/j.braindev.2019.12.010

[29] Kitaura H , Marahleh A , Ohori F , et al. Osteocyte‐related cytokines regulate osteoclast formation and bone resorption. Int J Mol Sci. 2020;21(14):5169. 32708317 10.3390/ijms21145169 PMC7404053

[30] Reyes‐Garcia R , Garcia‐Martin A , Garcia‐Fontana B , et al. FGF23 in type 2 diabetic patients: relationship with bone metabolism and vascular disease. Diabetes Care. 2014;37(5):e89‐e90. 24757249 10.2337/dc13-2235

[31] Mirza MA , Karlsson MK , Mellstrom D , et al. Serum fibroblast growth factor‐23 (FGF‐23) and fracture risk in elderly men. J Bone Miner Res. 2011;26(4):857‐864. 20928885 10.1002/jbmr.263

[32] Wei X , Huang X , Liu N , Qi B , Fang S , Zhang Y . Understanding the stony bridge between osteoporosis and vascular calcification: impact of the FGF23/Klotho axis. Oxidative Med Cell Longev. 2021;2021:7536614. 10.1155/2021/7536614 PMC8448600 34539972

[33] Fukumoto S . [Hormones and osteoporosis update. FGF23/Klotho and bone metabolism]. Clin Calcium. 2009;19(7):945‐950. 19567989

[34] Yokota H , Raposo JF , Chen A , Jiang C , Ferreira HG . Evaluation of the role of FGF23 in mineral metabolism. Gene Regul Syst Bio. 2009;3:131‐142. 10.4137/grsb.s2990 PMC2758275 19838340

[35] Brzeczek M , Hyla‐Klekot L , Kokot F , et al. Contribution of bone tissue to regulation of calcium and phosphate metabolism. Role of FGF23 and Klotho protein. Ortop Traumatol Rehabil. 2020;22(2):69‐76. 32468993 10.5604/01.3001.0014.1153

[36] Takeshita A , Kawakami K , Furushima K , Miyajima M , Sakaguchi K . Central role of the proximal tubular alphaKlotho/FGF receptor complex in FGF23‐regulated phosphate and vitamin D metabolism. Sci Rep. 2018;8(1):6917. 29720668 10.1038/s41598-018-25087-3 PMC5932018

[37] Bowden J , Davey SG , Burgess S . Mendelian randomization with invalid instruments: effect estimation and bias detection through egger regression. Int J Epidemiol. 2015;44(2):512‐525. 26050253 10.1093/ije/dyv080 PMC4469799

[38] Bowden J , Del GMF , Minelli C , et al. A framework for the investigation of pleiotropy in two‐sample summary data Mendelian randomization. Stat Med. 2017;36(11):1783‐1802. 28114746 10.1002/sim.7221 PMC5434863

[39] Burgess S , Bowden J , Fall T , Ingelsson E , Thompson SG . Sensitivity analyses for robust causal inference from Mendelian randomization analyses with multiple genetic variants. Epidemiology. 2017;28(1):30‐42. 27749700 10.1097/EDE.0000000000000559 PMC5133381

[40] Quarles LD . Role of FGF23 in vitamin D and phosphate metabolism: implications in chronic kidney disease. Exp Cell Res. 2012;318(9):1040‐1048. 22421513 10.1016/j.yexcr.2012.02.027 PMC3336874

[41] Feng JQ , Clinkenbeard EL , Yuan B , White KE , Drezner MK . Osteocyte regulation of phosphate homeostasis and bone mineralization underlies the pathophysiology of the heritable disorders of rickets and osteomalacia. Bone. 2013;54(2):213‐221. 23403405 10.1016/j.bone.2013.01.046 PMC3672228

[42] Liu S , Tang W , Fang J , et al. Novel regulators of Fgf23 expression and mineralization in Hyp bone. Mol Endocrinol. 2009;23(9):1505‐1518. 19556340 10.1210/me.2009-0085 PMC2737552

[43] Kuro‐O M , Matsumura Y , Aizawa H , et al. Mutation of the mouse Klotho gene leads to a syndrome resembling ageing. Nature. 1997;390(6655):45‐51. 9363890 10.1038/36285

[44] Kurosu H , Yamamoto M , Clark JD , et al. Suppression of aging in mice by the hormone Klotho. Science. 2005;309(5742):1829‐1833. 16123266 10.1126/science.1112766 PMC2536606

[45] Raimann A , Ertl DA , Helmreich M , Sagmeister S , Egerbacher M , Haeusler G . Fibroblast growth factor 23 and Klotho are present in the growth plate. Connect Tissue Res. 2013;54(2):108‐117. 23206185 10.3109/03008207.2012.753879

