# Supplementary figures and images for: Fibroblast growth factor 23‐mediated regulation of osteoporosis: Assessed via Mendelian randomization and in vitro study
Source: J Cell Mol Med. 2024 Jul 25;28(14):e18551. doi: 10.1111/jcmm.18551 (PMC11272609; doi:10.1111/jcmm.18551)

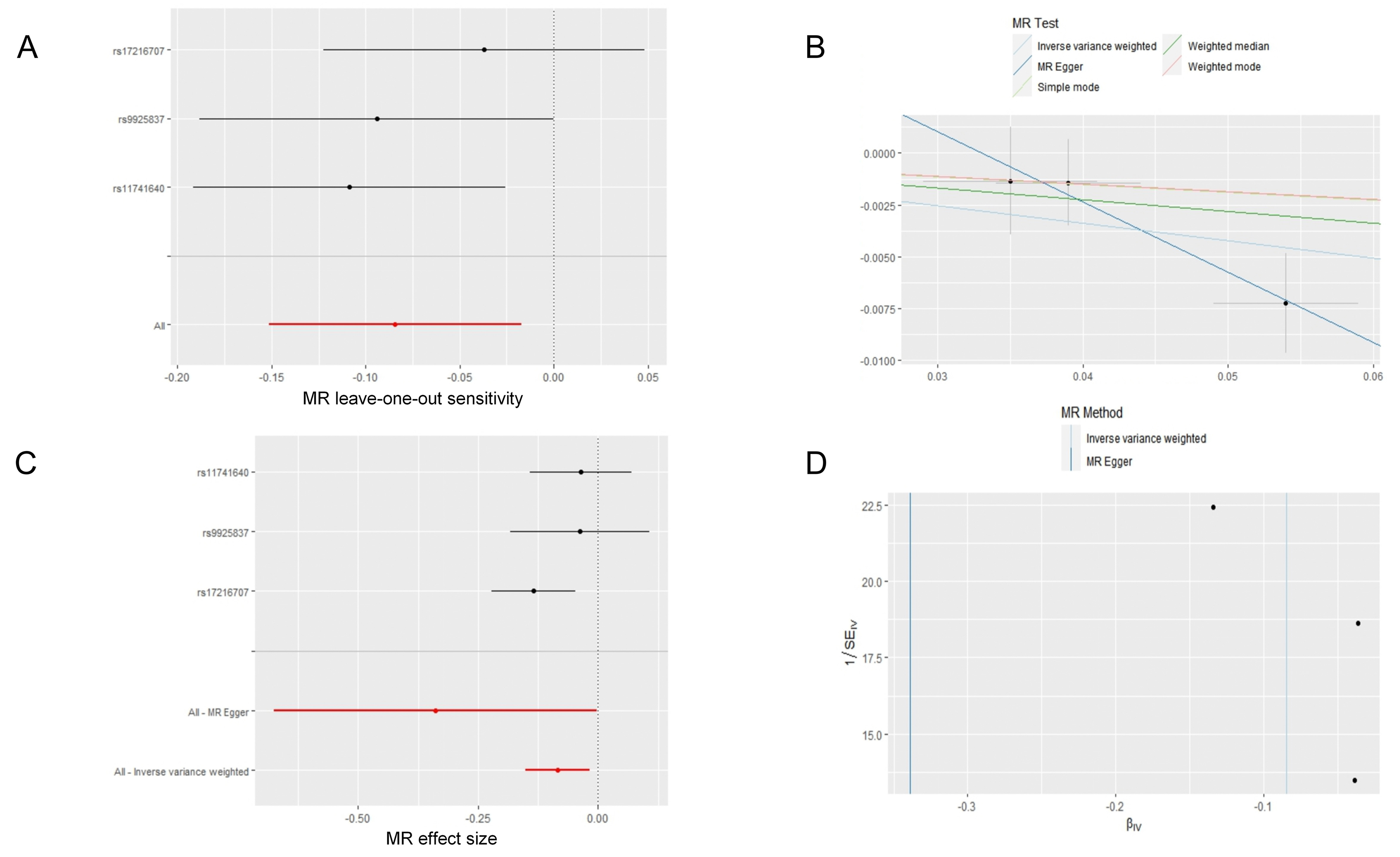

Supplement: Supplementary file 1 — Figure S1. [file JCMM-28-e18551-s001.jpg]

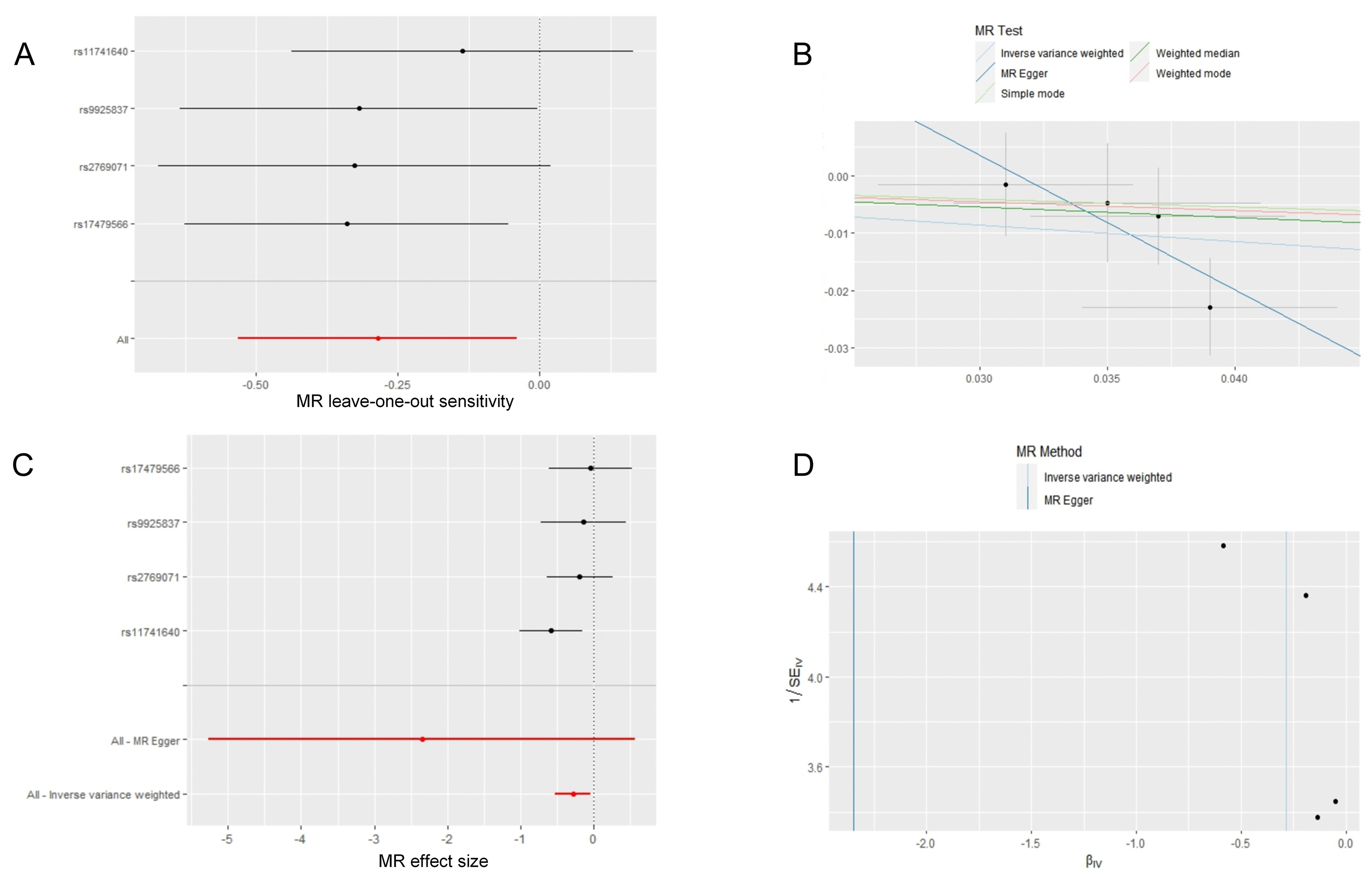

Supplement: Supplementary file 2 — Figure S2. [file JCMM-28-e18551-s003.jpg]
