# Supplementary material for: Fibroblast growth factor 23‐mediated regulation of osteoporosis: Assessed via Mendelian randomization and in vitro study
Source: J Cell Mol Med. 2024 Jul 25;28(14):e18551. doi: 10.1111/jcmm.18551 (PMC11272609; doi:10.1111/jcmm.18551)
Supplement: Supplementary file 3 — Table S1. Table S2. Table S3. Table S4. Table S5. Table S6. Table S7. [file JCMM-28-e18551-s002.docx]

**Supplementary Material**

**Table of Contents**

Supplementary Table S1: FGF23 instruments used in the MR analysis.…………………2

Supplementary Table S2: α-Klotho instruments used in the MR analysis.……………….3

Supplementary Table S3: FGFR1 instruments used in the MR analysis.………………...4

Supplementary Table S4: Information of the data sources for outcomes.………………..5

Supplementary Table S5: Characteristics of patients with osteoporotic fractures and non-osteoporotic fractures group.…………………......................................................6

Supplementary Table S6: The sequence of PCR primers in this study.…………………..7

Supplementary Table S7: Sensitivity MR analyses.…………….….….….….….….….…8

**Supplementary Table S1: FGF23 instruments used in the MR analysis.**

| **SNP** | **Chr** | **Position (hg19)** | **Gene** | **EA** | **OA** | **EAF** | **β** | **SE** | **P-value** | **F** |
| --- | --- | --- | --- | --- | --- | --- | --- | --- | --- | --- |
| **rs17216707** | 20 | 54115823 | BCAS1/ CYP24A1 | T | C | 0.80 | 0.054 | 0.005 | 3.00×10^-24^ | 117 |
| **rs11741640** | 5 | 177365742 | RGS14 | G | A | 0.73 | 0.039 | 0.005 | 1.60×10^-16^ | 61 |
| **rs17479566** | 9 | 68583098 | TMEM252-DT | T | C | 0.22 | 0.031 | 0.005 | 2.00×10^-09^ | 38 |
| **rs9925837** | 16 | 79893406 | LINC01228/ DYNLRB2-AS1 | G | A | 0.13 | 0.035 | 0.006 | 5.10×10^-09^ | 34 |
| **rs2769071** | 9 | 133270565 | ABO | G | A | 0.37 | 0.037 | 0.005 | 6.10×10^-17^ | 55 |

Note: chr, chromosome; EA, effect allele; OA, other allele; EAF, effect allele frequency; SE, standard error.

**Supplementary Table S2: α-Klotho instruments used in the MR analysis.**

| **SNP** | **Chr** | **Position (hg19)** | **Gene** | **EA** | **OA** | **EAF** | **β** | **SE** | **P-value** | **F** |
| --- | --- | --- | --- | --- | --- | --- | --- | --- | --- | --- |
| **rs881301** | 8 | 38332318 | LINC03042/FGFR1 | T | C | 0.587 | 0.118 | 0.021 | 2.23×10^-08^ | 32 |
| **rs8176672** | 9 | 136142185 | ABO | T | C | 0.071 | 0.406 | 0.041 | 2.11×10^-23^ | 98 |
| **rs532436** | 9 | 136149830 | ABO | A | T | 0.214 | -0.204 | 0.026 | 5.86×10^-15^ | 62 |
| **rs1056008** | 12 | 662838 | B4GALNT3 | T | C | 0.731 | -0.183 | 0.024 | 1.80×10^-14^ | 58 |
| **rs7333961** | 13 | 33533269 | KL/ TOMM22P3 | A | G | 0.046 | -0.327 | 0.051 | 1.73×10^-10^ | 41 |
| **rs12607664** | 18 | 24693221 | AQP4-AS1/ CHST9 | T | G | 0.316 | 0.242 | 0.022 | 2.28×10^-27^ | 121 |

Note: chr, chromosome; EA, effect allele; OA, other allele; EAF, effect allele frequency; SE, standard error.

**Supplementary Table S3: α-Klotho instruments used in the MR analysis.**

| **SNP** | **Chr** | **Position (hg19)** | **Gene** | **EA** | **OA** | **EAF** | **β** | **SE** | **P-value** | **F** |
| --- | --- | --- | --- | --- | --- | --- | --- | --- | --- | --- |
| **rs17216707** | 20 | 52732362 | BCAS1/CYP24A1 | T | C | 0.80 | 0.054 | 0.005 | 3.00×10^-24^ | 117 |
| **rs11741640** | 5 | 176792743 | RGS14 | G | A | 0.73 | 0.039 | 0.005 | 1.60×10^-16^ | 61 |
| **rs17479566** | 9 | 71198013 | TMEM252-DT | T | C | 0.22 | 0.031 | 0.005 | 2.00×10^-09^ | 38 |
| **rs9925837** | 16 | 79927303 | LINC01228/ DYNLRB2-AS1 | G | A | 0.13 | 0.035 | 0.006 | 5.10×10^-09^ | 34 |
| **rs2769071** | 9 | 136145974 | ABO | G | A | 0.37 | 0.037 | 0.005 | 6.10×10^-17^ | 55 |

Note: chr, chromosome; EA, effect allele; OA, other allele; EAF, effect allele frequency; SE, standard error.

**Supplementary Table S4: Information of the data sources for outcomes.**

|  | **Sample size** | **Year** | **SNP number** | **PMID/Bio-Rxiv** | **Authors** | **ID** | **Population** |
| --- | --- | --- | --- | --- | --- | --- | --- |
| **Heel-BMD** | 426,824 | 2019 | 13,705,641 | 30598549 | Morris JA(UK Biobank) | ebi-a-GCST006979 | European |
| **Femoral neck-BMD** | 32,735 | 2015 | 10,586,900 | 26367794 | Zheng(GEFOS) | ieu-a-980 | Mixed |
| **Lumbar spine-BMD** | 28,498 | 2015 | 10,582,867 | 26367794 | Zheng(GEFOS) | ieu-a-982 | Mixed |
| **Forearm-BMD** | 21,907 | 2020 | 11,312,319 | 33097703 | Surakka I(HUNT Study) | ebi-a-GCST90013422 | European |
| **Total body-BMD** | 56,284 | 2018 | 16,162,733 | 29304378 | Medina-Gomez C(GEFOS) | ebi-a-GCST005348 | European |
| **Osteoporosis with patholo-gical fracture** | 173,619 | 2021 | 16,380,281 | https://gwas.mrcieu.ac.uk | GEFOS | finn-b-OSTEOPOROSIS_FRACTURE_FG | European |

**Supplementary Table S5: Characteristics of patients with osteoporotic fractures and non-osteoporotic fractures group.**

| **Variable** | **Total**  **(n=40)** | **osteoporotic fractures**  **(n=20)** | **non-osteoporotic fractures**  **(n=20)** | ***P*-Value^＋^** |
| --- | --- | --- | --- | --- |
| **Gender** |  |  |  |  |
| Male | 40(100%) | 20(100%) | 20(100%) |  |
| **Age** |  |  |  |  |
| 55-65 | 12(30%) | 8(40%) | 4(20%) |  |
| >=65 | 28(70%) | 12(60%) | 16(80%) |  |
| **Covariates** |  |  |  |  |
| Hypertension | 5(12.5%) | 3(15%) | 2(10%) | 0.723 |
| Diabetes | 6(15%) | 4(20%) | 2(10%) | 0.750 |
| BMI (kg/m^2^) |  | 23.47±3.40 | 24.42±3.12 | 0.611 |
| serum calcium (mmol/L) |  | 2.33±0.14 | 2.28±0.09 | 0.051 |
| serium phosphorus (mmol/L) |  | 1.12±0.18 | 1.32±0.19 | 0.791 |
| **Blood lipid profile** |  |  |  |  |
| TC (mmol/L) |  | 5.33±0.93 | 4.79±0.13 | 0.048 |
| TG (mmol/L) |  | 1.48±0.59 | 1.32±0.56 | 0.156 |
| LDL-c (mmol/L) |  | 3.45±0.85 | 3.01±0.79 | 0.002 |
| HDL-c (mmol/L) |  | 1.32±0.34 | 1.35±0.37 | 0.664 |

**Note:** BMI, body mass index; TC, total cholesterol; TG, triglyceride; LDL-c, low density lipoprotein cholesterol; HDL-c, high density lipoprotein cholesterol; ^＋^ all test used the χ^2^ test.

**Supplementary Table S6: The sequence of PCR primers in this study.**

| **Primer** | **Sequences** |
| --- | --- |
| FGF23 | Forward: 5′-ATGCTAGGGACCTGCCTTAGACTC-3′  Reverse:5′-AGTGATGCTTCTGCGACAAGTAGAC-3′ |
| α-Klotho | Forward: 5′-ACCAGCAACCCCACACCAAG-3′  Reverse:5′-CCGCACGGTATGTCATGTTC-3′ |
| FGFR1 | Forward: 5′--CTGGTATCCTGTGCCTATC--3′  Reverse:5′-CAATCTGATCCCAAGACCAC-3′ |
| RUNX2 | Forward: 5′-GTAGATGGACCTCGGGAACC-3′  Reverse: 5′-GAGGCGGTCAGAGAACAAAC-3′ |
| OCN | Forward: 5′-GCAATAAGGTAGTGAACAGACTCC-3′  Reverse: 5′-GTTTGTAGGCGGTCTTCAAGC-3′ |
| OSX | Forward: 5′-ATGGGCTCCTTTCACCTG-3′-  Reverse: 5′-GGGAAAAGGGAGGGTAATC-3′ |
| GAPDH | Forward: 5ʹ-TCCTCTGACTTCAACAGCGA-3’  Reverse: 5ʹ-GGGTCTTACTCCTTGGAGGC-3’ |

**Supplementary Table S7: Sensitivity MR analyses**

| **Exposure** | **Outcomes** | **n(SNPs)** | **Heterogeneity test** | | **Pleiotropy test** | |
| --- | --- | --- | --- | --- | --- | --- |
|  |  |  | **Q test (Q *P*-Value)** | **Multiplicative rand-om effect (P-Value)** | **MR-Egger intercept(*P*-Value)** | **MR-PRESSO global test(P-Value)** |
| **FGF23** | Heel-BMD | 3 | 0.152 (0.697) | / | 0.011 (0.373) | 0.089 |
|  | Femoral neck-BMD | 4 | 2.996 (0.392) | / | 0.074 (0.299) | 0.153 |
|  | Lumbar spine-BMD | 4 | 5.462 (0.141) | / | 0.093(0.356) | 0.164 |
|  | Forearm-BMD | 4 | 7.180 (0.066) | / | 0.011(0.872) | 0.227 |
|  | Total body-BMD | 4 | 0.583 (0.900) | / | 0.003 (0.870) | 0.094 |
|  | Osteoporosis with pat-hological fracture | 4 | 1.400(0.705) | / | 0.166(0.387) | 0.331 |
| **α-Klotho** | Heel-BMD | 4 | 16.957 (7.21×10^-4^) | -1.29×10^-3^(0.919) | -0.011 (0.094) | 0.546 |
|  | Femoral neck-BMD | 4 | 1.274 (0.735) | / | -9.22×10^-4^(0.945) | 0.056 |
|  | Lumbar spine-BMD | 4 | 4.770 (0.184) | / | -0.013(0.577) | 0.987 |
|  | Forearm-BMD | 5 | 1.352 (0.852) | / | -0.011(0.556) | 0.059 |
|  | Total body-BMD | 4 | 12.245 (6.62×10^-3^) | 0.033(0.289) | -0.025 (0.223) | 0.211 |
|  | Osteoporosis with pat-hological fracture | 5 | 6.466(0.167) | / | 0.062(0.587) | 0.159 |
| **FGFR1** | Heel-BMD | 4 | 6.161 (0.104) | / | 0.012 (0.547) | 0.289 |
|  | Femoral neck-BMD | 3 | 0.111 (0.946) | / | -0.040 (0.801) | 0.173 |
|  | Lumbar spine-BMD | 3 | 0.536 (0.765) | / | 0.004(0.982) | 0.247 |
|  | Forearm-BMD | 5 | 0.239 (0.993) | / | -0.007(0.886) | 0.336 |
|  | Total body-BMD | 4 | 1.919 (0.589) | / | 0.040 (0.351) | 0.414 |
|  | Osteoporosis with pat-hological fracture | 5 | 4.582(0.333) | / | -0.511(0.132) | 0.281 |

**Note:** Cochran's Q test was derived from the IVW estimates and used to explore potential heterogeneity between IVs. When significant heterogeneity (*P*< 0.05) was detected, the IVW model of multiplicative random effects were used to detect it. The MR-Egger regression-derived MR-Egger intercept and MR-PRESSO global test were used to examine directional pleiotropy of IVs, with *P*< 0.05 as the threshold for significant pleiotropy.
